# Essential Oral Single Nutritional Therapy Products for Inherited Metabolic Diseases: Evidence and Consensus Assessment Using a Modified Delphi Method

**DOI:** 10.1002/jimd.70252

**Published:** 2026-09-30

**Authors:** Nina N. Stolwijk, Bart M. F. Penninx, Annet M. Bosch, Mirjam Langeveld, Gajja S. Salomons, Carla E. M. Hollak, Essential Nutritional Therapy Products for IMDs Consensus Group, Dorothea Möslinger, Saskia Wortmann, David Cassiman, Pavel Ješina, Jiri Zeman, Allan M. Lund, François Feillet, Nazi Tabatadze, Sarah C. Grünert, Anke Schumann, Ute Spiekerkoetter, Ulrike Mütze, Thomas Opladen, Anastasia Skouma, Ina Knerr, Serena Gasperini, Vincenza Gragnaniello, Mariarosa (Marina) Anna Beatrice Melone, Francesca Nardecchia, Alessandro La Rosa, Birutė Tumienė, Margreet Wagenmakers, Terry Derks, Camilla Tøndel, Beata Kieć‐Wilk, Urh Groselj, Amaya Bélanger‐Quintana, Javier De las Heras, Mireia del Toro Riera, Eva Venegas Moreno, Matthias Gautschi, Johannes Häberle, Elaine Murphy

**Affiliations:** ^1^ Medicines for Society (Medicijn voor de Maatschappij) Platform at Amsterdam UMC—University of Amsterdam Amsterdam the Netherlands; ^2^ Department of Endocrinology and Metabolism. Amsterdam UMC, Amsterdam Gastroenterology Endocrinology Metabolism (AGEM) Research Institute, Expertise Center for Inborn Errors of Metabolism, MetabERN University of Amsterdam Amsterdam the Netherlands; ^3^ Department of Pediatrics, Division of Metabolic Diseases, Emma Children's Hospital, Amsterdam Gastroenterology Endocrinology Metabolism (AGEM) Amsterdam UMC Location University of Amsterdam Amsterdam the Netherlands; ^4^ Department of Laboratory Medicine, Laboratory Genetic Metabolic Diseases Amsterdam UMC Location University of Amsterdam Amsterdam the Netherlands

**Keywords:** Delphi consensus procedure, essential medicines, inherited metabolic diseases, nutritional therapy products, patient access

## Abstract

Nutritional therapy is critical in managing inherited metabolic diseases (IMDs), and includes specialized diets and single nutritional therapy products (sNTPs) such as vitamins, cofactors, and amino acids. Many sNTPs function as medicines, but are regulated as food (e.g., food supplements), which can limit access, reimbursement, and consistent supply. Essential medicines, as defined by World Health Organization (WHO), meet key healthcare needs and must be available, affordable, and quality‐assured. Some sNTPs might meet this threshold, supporting their potential inclusion in the WHO essential medicines list. A modified Delphi process was conducted with a panel of 36 experts from 18 European countries to identify which sNTPs are essential for IMD treatment. Ninety‐six sNTPs and their target IMDs were assessed using a literature‐informed five‐criteria framework, that included evaluation of the level of scientific evidence for usefulness and effectiveness. Overall, 31 sNTPs were deemed essential in the management of 55 IMDs, consisting of vitamins (*n* = 12), minerals (*n* = 4), amino acids (*n* = 6), sugars (*n* = 2), and other nutrients (*n* = 7). Evidence supporting their use ranged from levels 3 to 4 of the Oxford Centre for Evidence‐Based Medicine (OCEBM), reflecting the limited clinical trial data inherent to rare diseases. Seven of these are already included in the WHO essential medicines list. Recognition of the remaining 24 essential sNTPs could improve their availability and reimbursement, and in some cases, may warrant formal authorization as medicines. Subsequent research should focus on access inequities, cost barriers, and incorporate patient perspectives to optimize the use and impact of sNTPs in IMD management.

## 1 Introduction

Nutritional therapy is of critical importance in the management of many inherited metabolic diseases (IMDs) and can involve various approaches, including specialized diets, low‐protein foods, and single‐molecule nutritional therapy products (sNTPs). The latter category includes single amino acids, vitamins and cofactors [1]. Products used in nutritional therapy exist on the continuum from food to medicine, addressing patient needs that range from fulfilling dietary requirements to pharmacological intervention [1, 2]. Despite this spectrum, the majority of nutritional therapy products are regulated as food or food supplements rather than medicinal products—even if they *function* as medicine. The single amino acid citrulline, for example, is not authorized as medication, yet clinical guidelines categorize its use in urea cycle disorder (UCD) management as pharmacotherapy [3]. Regulating these products as food can impact their availability, affordability and suitability (i.e., quality and dosing), with potentially dire consequences: in one case, an infant with carbamoyl phosphate synthetase deficiency Type 1 (CPS1 deficiency, OMIM #237300) had a recurrence of hyperammonemia after using a product marketed as “99.88% pure l‐citrulline” which, upon testing, was found to lack the amino acid entirely [4].

In order to improve access to the nutritional therapy products required for adequate IMD treatment, there is a growing need to recognize those that are essential for the management of these conditions. The WHO defines essential medicines as those that address key healthcare needs and should be available at all times, within functioning health systems, in adequate amounts, in appropriate dosage forms, with assured quality, and at affordable prices [5]. This concept, when applied to IMD treatments, emphasizes the need for certain sNTPs to be classified as essential in order to improve their availability, affordability, and suitability. This study aims to identify nutritional therapeutic products that function as medicines and to determine which are essential for the management of IMDs, based on international expert consensus.

## 2 Methods

To achieve consensus on which sNTPs are considered essential, a modified Delphi procedure was employed. Input was derived from a database of sNTPs compiled through a literature review and expert opinion. While the classical Delphi method involves multiple rounds of questionnaires to reach consensus, the modified approach commenced with a structured questionnaire based on existing literature, reducing the number of rounds required [6, 7].

### 2.1 Expert Panel

All metabolic physicians with membership of the European Reference Network for Hereditary Metabolic Disorders [8] (MetabERN) were invited to join the expert panel. To further encourage participation, a number of MetabERN members were additionally contacted personally (*n* = 30). These were metabolic physicians with recognized expertise in specific disorder groups defined by active clinical practice and substantial research contributions within the respective disorder group, selected by the study team. Seven personal invitees did not respond and two were unable to participate due to scheduling constraints. The final panel included 36 experts representing 30 centers across 18 European countries: Austria (*n* = 2), Belgium (*n* = 1), the Czech Republic (*n* = 2), Denmark (*n* = 1), France (*n* = 1), Georgia (*n* = 1), Germany (*n* = 5), Greece (*n* = 1), Ireland (*n* = 1), Italy (*n* = 5), Lithuania (*n* = 1), the Netherlands (*n* = 5), Norway (*n* = 1), Poland (*n* = 1), Slovenia (*n* = 1), Spain (*n* = 4), Switzerland (*n* = 2), and the United Kingdom (*n* = 1).

### 2.2 Defining sNTPs

Nutritional treatment for IMDs consists of several different types of approaches, ranging from diets restricted and/or enriched in specific nutrients to the use of specifically formulated amino‐acid mixtures, low‐protein food and single amino acids or vitamins. There is no consensus on terminology or classification of these varying strategies. In this study, the focus is on *products* prescribed. Therefore, diets that exclude or reduce the intake of certain nutrients fall outside the scope. For nutritional therapy products, the following categorization has been proposed: (1) bulk nutritional products formulated to omit a disease‐relevant amino acid (e.g., phenylalanine free amino acid mixtures for PKU), (2) low‐protein foods, (3) (single) nutrients to replace conditionally essential nutrients or enhance enzyme activity (e.g., biotin for biotinidase deficiency) [1]. This study focuses on the single‐molecule NTPs (sNTPs) in the third category, defined here as nutrients and other food‐related substances with a single molecule as the active ingredient that could theoretically be authorized as medicinal products. Products not intended for oral ingestion do not fall within the regulatory definition of food and are therefore regulated as medicinal products [2, 9, 10]. Their status as medicines is not in question and they therefore fall outside the scope of this study, which aims to discern which sNTPs at the intersection of food and medicine could be considered essential medication.

### 2.3 Database Development and Literature Review

To compile a comprehensive list of food‐related treatments for IMDs, the standard textbook *Inborn Metabolic Diseases: Diagnosis and Treatment* (7th edition, 2022) [11] was used as a primary source. A database encompassing all IMDs mentioned in this textbook was created. The database included the following parameters for each disorder: name, disorder group, gene(s) involved, described treatments and whether these included nutritional treatment products (yes/no). For each sNTP, the active ingredient(s) were also extracted. To verify disease classification and gene–disease associations, all disorders mentioned in the textbook were cross‐referenced with the Online Mendelian Inheritance in Man (OMIM) database.

Subsequently, all sNTP–IMD pairs were extracted and divided between two primary reviewers (N.N.S. and B.M.F.P.), each independently reviewing an assigned subset of combinations. For each combination, a targeted literature search was performed in PubMed combining the disease name and sNTP active ingredient (including relevant abbreviations and/or variations) as primary search terms. A second targeted search using the same terms combined with “guideline” or “consensus” was performed to identify relevant clinical practice recommendations. Searches were conducted between July 2024 and January 2025. Only articles with full‐text available in English were included in the review. Instead of systematic screening of reference lists, the cited primary studies that were directly relevant to the specific sNTP–IMD pair were selectively examined and included. Conference abstracts were excluded. Relevant regulatory documents from the European Food Safety Authority (EFSA), such as relevant scientific opinions on Dietary Reference Values (DRVs), were consulted separately.

The relevant literature was evaluated based on our previously published flowchart [2] (see Figure 1), to delineate essential, medication‐like sNTPs. Reviewers met regularly to discuss assessments and resolve discrepancies. Prior to full‐scale assessment, the methodology was calibrated through discussion of an initial subset of sNTP–IMD pairs with the full study team (C.E.M.H., A.M.B., G.S.S., N.N.S., and B.M.F.P). Upon completion, the entire list of inclusions and exclusions was reviewed and approved by the remaining study team members (C.E.M.H., A.M.B., and G.S.S.).

**FIGURE 1:**
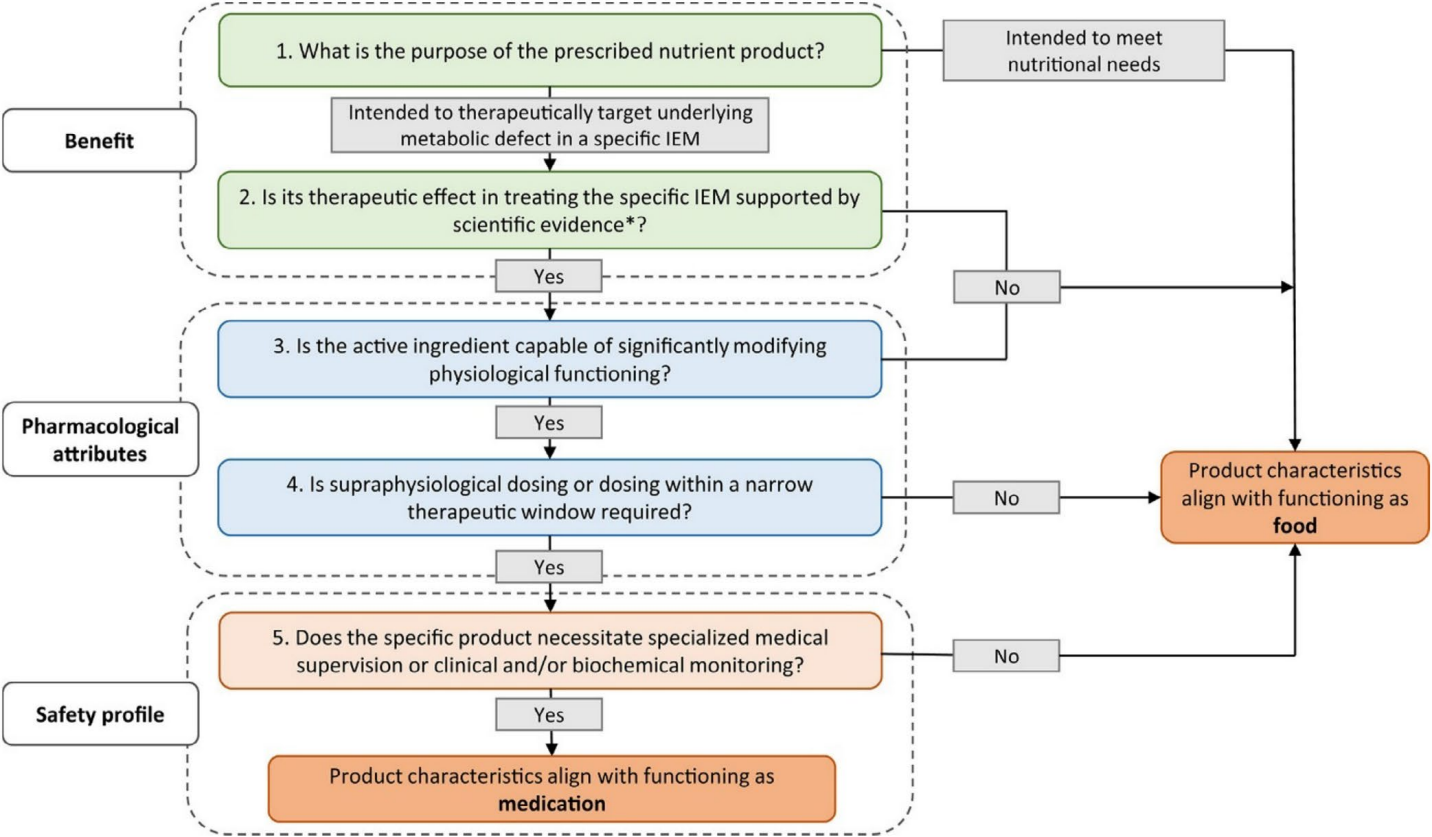
Proposed flowchart for distinguishing between food and medication‐like functioning of single‐molecule nutritional therapy products. Reproduced from Stolwijk et al. [2]. *Please note that this flowchart does not address the quality of scientific evidence supporting the therapeutic effect of the product. Nevertheless, it is important to acknowledge that consideration of the level of evidence may be relevant and examined in potential subsequent stages, such as during an application for marketing authorization.

### 2.4 Defining the Functioning of sNTPs: A Case‐Based Approach

Five elements from our previously published flowchart [2] for distinguishing between food and medication‐like functioning of sNTPs were assessed: (1) purpose; (2) scientific evidence; (3) physiological functioning of the active ingredient; (4) dosing; and (5) monitoring requirements (see Figure 1). Primary reviewers (N.N.S. and B.M.F.P.) performed the assessments with periodic expert review by senior clinicians (M.L., A.M.B., G.S.S., and C.E.M.H.). The elements were operationalized as follows:

#### 2.4.1 Purpose

The first criterion defined the aim of the sNTP: either to meet nutritional needs or to therapeutically target the underlying defect. To clarify the purpose of the sNTP for each specific IMD, the mechanism of action as described in the literature was evaluated against the pathophysiological mechanism to determine if its aim was to directly target the metabolic defect. If the mechanism of action did directly target the metabolic defect, the item was rated as “yes.” If it addressed nutritional needs or provided additional energy, it was rated as “no.” sNTPs that did not directly target the defect were excluded from further analysis. In cases where the pathophysiological mechanism was poorly understood and the link between the action mechanism and the defect was unclear, the purpose was rated as “unclear” and excluded.

#### 2.4.2 Scientific Evidence

The second criterion assessed the scientific evidence to evaluate if there are sufficient data to support the sNTPs role in treating or managing the condition. To this end, the Oxford Centre for Evidence‐Based Medicine (OCEBM) levels of evidence (2011 version) were used to grade the scientific evidence for sNTP‐IMD pairs [12]. In this system, Level 1 is assigned to systematic reviews of randomized (n‐of‐1) trials, Level 2 to randomized trials or observational studies with dramatic effect, Level 3 to non‐randomized controlled cohort studies, Level 4 to non‐analytical (case report/series) and historically controlled studies and Level 5 for mechanism‐based reasoning. Downgrading of levels can occur based on factors including low quality studies and/or inconsistent reporting, while upgrading is possible in case of a (very) large effect size. Only sNTPs supported by evidence graded ≤ Level 4 were included in the Delphi process.

#### 2.4.3 Physiological Functioning

For this third criterion, the sNTP's ability to significantly modify physiological functioning was assessed by reviewing the mechanism of action described in the literature. According to the case‐law of the Court of Justice of the European Union, substances that do not exert a significant physiological effect and do not, in a strict sense, modify bodily functions cannot be classified as medicinal products [2, 13].

#### 2.4.4 Dosing

For this fourth criterion, the dosing of the sNTP during the disease stage with the highest dosing requirements was evaluated by comparing nutrient intake in the general population with that prescribed in the specific IMD, to assess if dosing is supra‐physiological or within narrow therapeutic window and thereby medication‐like. Supraphysiologic doses were defined as quantities of nutrients that significantly exceed the levels typically found in a normal diet and cannot be attained through a small dietary modification. Reference intake levels were based on EU DRVs from EFSA where available. If no DRV existed, reported median intakes from general population cohorts were used and where neither was available, the nutrient content of a representative food item rich in the respective sNTP was analyzed to evaluate whether the prescribed dose could feasibly be met through diet alone. A narrow therapeutic window refers to a small range between the minimum effective dose and the minimum toxic dose of the sNTP.

#### 2.4.5 Monitoring Requirements

For the final criterion, the necessity for specialized medical supervision or clinical and/or biochemical monitoring related to the sNTP in the context of a specific IMD was evaluated. Monitoring might be required for several reasons, for example the risk of potential side effects (i.e., high risk of occurrence or risk of serious adverse events), risk of suboptimal therapeutic effectiveness (e.g., when the reported effective doses varied widely) and expected problems regarding compliance.

### 2.5 Modified Delphi Study

sNTPs with at least some observational evidence supporting their role in an inborn metabolic disorder (IMD) (≤ Level 4 evidence) and meeting all five flowchart criteria were deemed to fulfill criteria of potentially essential medicines and therefore included in the Delphi procedure. These sNTP–IMD combinations were compiled into an online survey using the web‐based tool eDelphi (https://www.edelphi.org, see Supporting Information File S2 for the structured questionnaires for round 1 and 2). Panel members were asked to rate each sNTP's essentiality for managing the associated IMD using a 3‐point Likert scale (agree, neutral, disagree). To standardize interpretation, panel members were provided with the WHO definition of essential medicines on each page of the survey (“*those that meet priority health care needs and should always be available in sufficient quantities, appropriate forms, assured quality and at affordable prices* [5]”) and were asked to apply this concept when judging the sNTP–IMD combinations. In addition, they could also indicate a lack of expertise regarding a specific disorder and not give a judgment.

Round 1 responses included both quantitative ratings and anonymous free‐text comments. Participants could provide justification for their ratings and suggest additional items. However, all comments were anonymized. Following the first round, responses were analyzed using Excel and R (version 4.3.1). Consensus for a sNTP being “essential” was defined as ≥ 75% agreement; combinations with ≥ 50% disagreement were excluded as “not essential.” Responses from panelists who indicated no expertise were excluded from the respective analyses. Items lacking consensus were re‐assessed in a second survey round. Additionally, newly proposed sNTPs during round one were screened for duplication and eligibility based on the original inclusion criteria (i.e., meeting the five flowchart criteria with ≤ Level 4 evidence) by the study team (N.N.S. and B.M.F.P.), with independent review (by A.M.B., M.L., G.S.S. and C.E.M.H.), prior to inclusion in round 2. After the second round, consensus was reassessed using the same predefined criteria. Combinations that were deemed neither “essential” nor “not essential” were categorized as “no consensus.” Data collection occurred between 28 February and 30 April 2025.

## 3 Results

A total of 165 sNTP‐IMD combinations were extracted from *Inborn Metabolic Diseases: Diagnosis and Treatment* [11] and were evaluated using the five flowchart criteria. Full disorder‐specific bibliographic documentation for all sNTP–IMD combinations is provided in Supporting Information File S1. Of these, 82 sNTP‐IMD combinations were included for round one of the Delphi procedure, comprising of 34 separate sNTPs applied for 59 IMDs. Several IMDs were linked to two or more sNTPs, which resulted in the 82 combinations included (see Figure 2. for an overview and Supporting Information File S1. for the full list). After round one, 29 additional suggestions for sNTP‐IMD combinations by panel members were analyzed using the flowchart criteria. This set comprised 17 sNTPs, including 8 not previously assessed in round one, and 23 IMDs, 20 of which had not been included in round one. Of these 29 panelist‐suggested sNTP‐IMD combinations, 14 met the criteria for inclusion in the Delphi procedure and were included for assessment in round two. This means that in total, 96 sNTP‐IMD combinations (consisting of 42 separate sNTPs for 79 IMDs) were evaluated in the Delphi procedure to determine the essentiality of the sNTP in the management of the target IMD.

**FIGURE 2:**
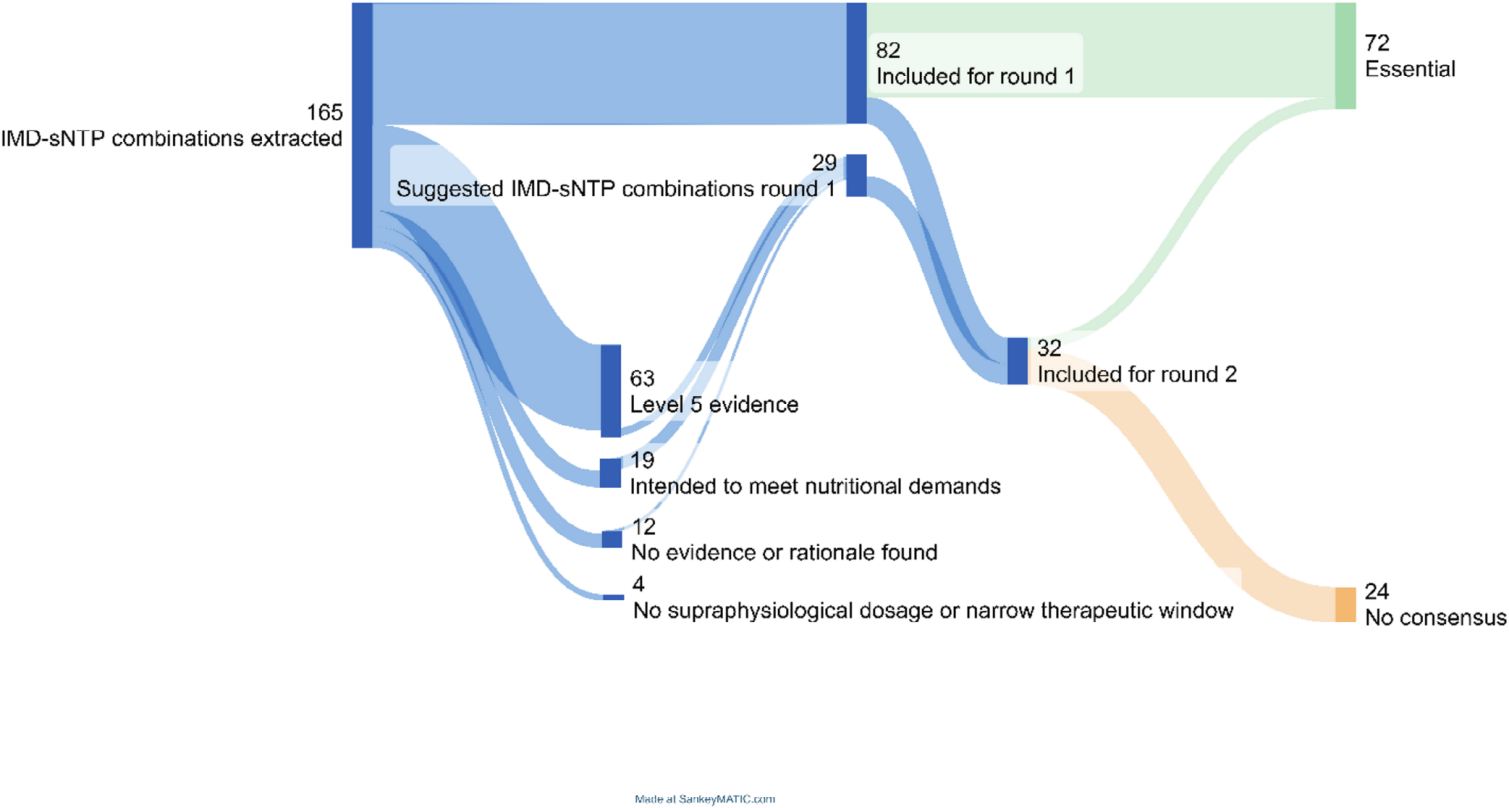
Selection and consensus outcomes for sNTP–IMD combinations. Sankey diagram showing categorization of 165 extracted sNTP‐IMD combinations and their progression through two Delphi rounds to final classification as “essential” or “no consensus.” “Essential” was judged according to the WHO essential medicines definition: Medicines that meet priority health care needs and should always be available in sufficient quantities, appropriate dosage forms, assured quality, and at affordable prices [5]. IMD, inherited metabolic disease; sNTP, single‐molecule nutritional therapy product.

Of the included combinations, around one‐third (*n* = 29, 30%) were supported by Level 3 evidence. Some received this classification based on larger observational studies using prospectively collected data (e.g., sodium benzoate for the management of several UCDs [14]), while others were upgraded due to large effect sizes despite being based on case studies (see Supporting Information File S1). The remaining 67 combinations (70%) were based on Level 4 evidence. The majority of the exclusions of sNTP‐IMD combinations from the Delphi procedure were attributable to insufficient evidence: the available evidence was either graded as Level 5 (*n* = 63), or no evidence or rationale could be found (*n* = 12). Other reasons for exclusion from the Delphi procedure related to the sNTP's purpose (*n* = 12) or dosage (*n* = 4) in the target IMD (see Figure 2).

### 3.1 Essential sNTPs

Thirty‐six panelists responded in round one of the Delphi procedure, and 35 participated in round two. Following both rounds, a total of 72 sNTP‐IMD combinations were deemed essential, consisting of 31 separate sNTPs applied in the management of 55 IMDs (see Table 1). Again, for some IMDs more than one sNTP was considered essential; CPS1 deficiency is an example, with arginine, citrulline, and sodium benzoate all classified as essential. The list of essential sNTPs for IMD management included 12 vitamins, four minerals, six amino acids, two sugars, and seven other nutrients. The expert panel classified all 29 sNTP‐IMD combinations with Level 3 evidence as essential. The use of the other 43 sNTP‐combinations deemed essential was supported by Level 4 evidence.

**TABLE 1:** NTPs identified as essential for managing corresponding IMDs.

Type	NTP	IMD(s)	Level of evidence^a^	Gene(s)	OMIM
Vitamins	Biotin	Biotinidase Deficiency	Level 3	*BTD*	#253260
		Holocarboxylase Synthetase Deficiency	Level 3	*HLCS*	#253270
		Sodium‐Dependent Multivitamin Transporter Deficiency	Level 4	*SLC5A6*	#618973
	Folate	Hereditary Folate Malabsorption (Proton‐Coupled Folate Transporter Deficiency)	Level 4	*SLC46A1*	#229050
	Folinic acid	Cerebral Folate Deficiency (Folate Receptor α Deficiency)	Level 4	*FOLR1*	#613068
		Dihydrofolate Reductase Deficiency	Level 4	*DHFR*	#613839
		Methylenetetrahydrofolate Dehydrogenase Deficiency	Level 4	*MTHFD1*	#617780
	Hydroxocobalamin	cblC	Level 3	*MMACHC, PRDX1*	#277400
		Methylmalonic aciduria (MMA)	Level 4	*MMUT, MMAA and MMAB*	#251100, #251110, #251120
	Nicotinamide	NAD(P)HX System Repair Defects	Level 4	*NAXE, NAXD*	#617186, #618321
	Pyridoxal phosphate	PLP Binding protein (PLPBP, Formerly PROSC) Deficiency	Level 4	*PLPBP*	#617290
		Pyridox(am)ine 5′‐phosphate Oxidase (PNPO) Deficiency	Level 3	*PNPO*	#610090
	Pyridoxine	Antiquitin Deficiency	Level 3	*ALDH7A1*	#266100
		Cystathionine β‐Synthase Deficiency	Level 3	*CBS*	#236200
		Ornithine Aminotransferase Deficiency (Gyrate Atrophy of the Choroid and Retina)	Level 4	*OAT*	#258870
		PLP Binding protein (PLPBP, Formerly PROSC) Deficiency	Level 4	*PLPBP*	#617290
		Primary hyperoxaluria Type I	Level 4	*AGXT*	#259900
		Pyridox(am)ine 5′‐phosphate Oxidase (PNPO) Deficiency	Level 3	*PNPO*	#610090
		X‐Linked sideroblastic anaemia	Level 4	*ALAS2*	#300751
	Riboflavin	Flavin adenine dinucleotide (FAD) Synthase deficiency	Level 4	*ACAD9*	#611126
		Mitochondrial FAD transporter deficiency	Level 4	*FLAD1*	#616839
		Multiple Acyl‐CoA Dehydrogenase Deficiency (MADD)	Level 3	*ETFA, ETFB, ETFDH*	#231680
		Riboflavin Transporter Deficiencies	Level 3	*SLC52A2 and SLC52A3*	#614707, #211530
	Thiamine	Thiamine Metabolism Dysfunction Syndrome 1 (THTR1 Deficiency)	Level 4	*SLC19A2*	#249270
		Thiamine Metabolism Dysfunction Syndrome 2: SLC19A3 Deficiency	Level 3	*SLC19A3*	#607483
		Thiamine Metabolism Dysfunction Syndrome 3 and 4: Mitochondrial TPP Transporter deficiency	Level 4	*SLC25A19*	#613710; #607196
		Pyruvate Dehydrogenase Complex (PDHC) Deficiency	Level 4	*PDHA1, PDHB*	#312170, #614111
	Vitamin C	Glutathione Synthetase Deficiency	Level 4	*GSS*	#266130
	Vitamin D	Cystinosis	Level 4	*CTNS*	#219800; #219900; # 219800; #219750
		Primary Hypomagnesaemia with Secondary Hypocalcaemia	Level 4	*TRPM6*	#602014
		X‐linked hypophosphatemic rickets, Autosomal recessive hypophosphatemic rickets, Autosomal dominant hypophosphatemic rickets	Level 4	*PHEX, DMP1, ENPP1, FGF23*	#307800, #241520, #613312, #193100
	Vitamin E	Glutathione Synthetase Deficiency	Level 4	*GSS*	#266130
Minerals	Magnesium	Primary Hypomagnesaemia with Secondary Hypocalcaemia	Level 4	*TRPM6*	#602014
	Phospate	X‐linked hypophosphatemic rickets	Level 4	*PHEX, DMP1, ENPP1, FGF23*	#307800, #241520, #613312, #193100
	Potassium	Cystinosis	Level 4	*CTNS*	#219800; #219900; #219800; #219750
	Zinc	Acrodermatitis Enteropathica	Level 3	*SLC39A4*	#201100
		Wilson Disease	Level 3	*ATP7B*	#277900
Amino acids	Arginine	Argininosuccinate lyase (ASL) deficiency	Level 3	*ASL*	#207900
		Carbamoyl phosphate synthetase 1 (CPS1) deficiency	Level 4	*CPS1*	#237300
		Citrin Deficiency	Level 4	*SLC25A13*	#603471, #605814
		Ornithine transcarbamylase (OTC) deficiency	Level 4	*OTC*	#311250
	Citrulline	Carbamoyl phosphate synthetase 1 (CPS1) deficiency	Level 3	*CPS1*	#237300
		Hyperornithinaemia, Hyperammonaemia and Homocitrullinuria (HHH) Syndrome	Level 4	*SLC25A15*	#238970
		Lysinuric protein intolerance	Level 4	*SLC7A7*	#222700
		Ornithine transcarbamylase (OTC) deficiency	Level 3	*OTC*	#311250
	Isoleucine	Maple syrup urine disease (MSUD)	Level 3	*BCKDHA, BCKDHB, DBT*	#248600, #620698, #620699
	Ornithine	Guanidinoacetate Methyltransferase (GAMT) Deficiency	Level 4	*GAMT*	#612736
	Serine	3‐Phosphoglycerate Dehydrogenase Deficiency	Level 4	*PHGDH*	#601815
	Valine	Maple syrup urine disease (MSUD)	Level 3	*BCKDHA, BCKDHB or DBT*	#248600, #620698, #620699
Sugars	Galactose	Phosphoglucomutase 1 ‐congenital disorder of glycosylation (PGM1‐CDG)	Level 3	*PGM1*	#614921
	Mannose	Mannose phosphate isomerase‐congenital disorder of glycosylation (MPI‐CDG)	Level 3	*MPI*	#602579
Other	5‐hydroxytryptophan	6‐pyruvoyl‐tetrahydropterin synthase deficiency	Level 4	*PTS*	#261640
	Betaine	CblC	Level 4	*MMACHC, PRDX1*	#277400
		Cystathionine β‐Synthase Deficiency	Level 4	*CBS*	#236200
		Methylenetetrahydrofolate Reductase Deficiency	Level 3	*MTHFR*	#236250
	Carnitine	Carnitine Transporter Deficiency	Level 3	*SLC22A5*	#212140
		Glutaric Aciduria Type I (Glutaryl‐CoA Dehydrogenase Deficiency)	Level 3	*GCDH*	#231670
		Malonyl‐CoA Decarboxylase Deficiency	Level 4	*MLYCD*	#248360
		Methylmalonic aciduria (MMA)	Level 4	*MMAA and MMAB MCEE*	#251100, #251110, #251120
		Propionic aciduria (PA)	Level 4	*PCCA or PCCB*	#606054
	Coenzyme Q10	Primary coenzyme Q10 deficiency Type 1	Level 4	*COQ2*	#607426
		Primary coenzyme Q10 deficiency Type 9	Level 4	*COQ5*	#619028
	Creatine‐monohydrate	Arginine: Glycine Amidinotransferase (AGAT) Deficiency	Level 4	*GATM*	#612718
		Guanidinoacetate Methyltransferase (GAMT) Deficiency	Level 3	*GAMT*	#612736
	Sodium benzoate	Argininosuccinate lyase (ASL) deficiency	Level 3	*ASL*	#207900
		Argininosuccinate synthetase (ASS) deficiency	Level 3	*ASS1*	#215700
		Carbamoyl phosphate synthetase 1 (CPS1) deficiency	Level 3	*CPS1*	#237300
		Hyperornithinaemia, Hyperammonaemia and Homocitrullinuria (HHH) Syndrome	Level 3	*SLC25A15*	#238970
		Ornithine transcarbamylase (OTC) deficiency	Level 3	*OTC*	#311250
	Uridine	Carbamoyl phosphate synthetase, Aspartate transcarbamylase, Dihydroorotase (CAD) deficiency	Level 4	*CAD*	#616457

^a^ Based on the Oxford Centre for Evidence‐Based Medicine (OCBEM) levels of evidence (2011 version) [12].

Essential sNTPs were accepted with a median agreement of 93% (IQR 86%–97%), while a median of 5% (IQR 3%–11%) remained neutral and 0% (IQR 0%–3%) disagreed. These agreements were reached by a median of 29 panelists (IQR 21–32), excluding a median of 7 (IQR 4–14) who indicated the question was outside their expertise. Overall, disagreement within the panel for accepted sNTPs was low: only four sNTP–IMD combinations classified as essential had more than one panelist who considered the sNTP non‐essential. These were arginine for ornithine transcarbamylase deficiency (OMIM #311250) and citrin deficiency (OMIM #603471, #605814), sodium benzoate for arginase‐1 deficiency (OMIM #207800), and folate for hereditary folate malabsorption (OMIM #229050).

### 3.2 sNTPs of Uncertain Essentiality

For 24 sNTP‐IMD combinations, no consensus was achieved on whether the sNTP should be considered essential in the management of the target IMD (see Supporting Information File S1). These sNTP‐IMD combinations were assessed by a panel of median 29 (IQR 22–31) in round 2 of the Delphi procedure, while a median of six (IQR 4–14) panelists indicated that the question was outside their expertise. It is notable that even these combinations without consensus still had a median of 52% of panelists (IQR 38%–61%) rating the sNTP essential to the target IMD, while 38% (IQR 29%–43%) remained neutral, and only 10% (IQR 7%–16%) judged them non‐essential.

For five sNTP‐IMD combinations (five sNTPs used in four IMDs) where no consensus was reached, over 50% of the panel indicated the question was outside their expertise. The IMDs involved were all ultra‐rare: one had an estimated prevalence below 1:1 000 000 (primary hypomagnesaemia with secondary hypocalcaemia, OMIM #602014), and the other three IMDs were described only in isolated case reports (methionyl‐tRNA synthetase deficiency, OMIM *156560, SLC35C1‐congenital disorders of glycosylation, OMIM*610804, and glutamate oxaloacetate transaminase, mitochondrial (GOT2) deficiency, OMIM #618721) [15, 16, 17, 18, 19, 20]. No sNTPs reached the threshold of ≥ 50% disagreement to be deemed as not essential for the target IMD.

## 4 Discussion

This study evaluated the sNTPs used in the management of IMDs to identify those functioning as medicines and considered essential by an expert panel. In total, 31 different sNTPs were identified as essential for use across 55 IMDs. In order to guarantee access to high quality, suitable forms of critically important sNTPs, these essential sNTPs should be made widely available at high quality and should be reimbursed. Regulating sNTPs as food instead of medicine means these products are less stringently regulated in areas such as quality control and safeguarding availability and reimbursement may be absent or insufficient [1, 2, 21]. In order to improve the availability and affordability of sNTPs, recognizing their essential role may be an important first step. The WHO essential medicines list is often used as a benchmark for procurement and reimbursement decisions worldwide, and it is our aim that this consensus‐based list of essential sNTPs could similarly be used to improve reimbursement and optimize access to these sNTPs for patients with IMDs.

Of the 31 sNTPs classified as essential, the current WHO essential medicines list does already include the following seven: ascorbic acid, calcium, colecalciferol, nicotinamide, pyridoxine, riboflavin, and thiamine [5]. Their listed indications, however, remain broad, such as calcium deficiency, and do not differentiate between inherited and acquired forms. Because the WHO list prioritizes medicines on the basis of disease prevalence and overall public health impact, it may not be the most appropriate mechanism for supporting IMD treatment access [22]. It has been proposed that a complementary list for rare diseases should be established, although the number of orphan medicinal products added to the WHO list has also increased in recent years [22, 23].

Current clinical guidelines for the relevant IMDs do already recommend 14 of the essential sNTPs for 26 target IMDs, underlining their established and important role in the best practice management of these disorders [3, 24, 25, 26, 27, 28, 29, 30, 31, 32, 33, 34]. Inclusion in the current WHO list may not be feasible or necessary for these products. Instead, the establishment of a separate, targeted list of essential treatment products for IMDs, modeled after the WHO list, may be a more practical solution. Incorporating not only sNTPs but also essential medicines into this list could broaden its applicability. Such a list could be incorporated into existing European networks of expertise, such as MetabERN, or maintained through a dedicated EU infrastructure for rare (metabolic) diseases. To ensure the list remains current with evolving evidence and clinical practice, periodic review by a standing expert panel is recommended. This expanded list could serve as a reference point for national regulatory bodies in reimbursement decisions, as well as enable clinicians and patient organizations to substantiate reimbursement claims with a scientifically and expert‐backed basis to improve access to essential IMD treatment products.

For 24 sNTP‐IMD combinations, consensus on essentiality was not reached. Notably, even among these combinations, a median of 52% of panelists (IQR 38%–61%) did consider the sNTP essential. This may reflect the considerable heterogeneity of clinical practice in the management of many IMDs. For instance, the use of carnitine in long‐chain fatty acid oxidation disorders (LC‐FOADs) is generally not recommended, or only considered when free carnitine levels are low, according to expert opinion and guidelines [35, 36]. Nevertheless, survey data show that 17%–25% of metabolic physicians prescribe it for most or all of their patients with long‐chain hydroxyacyl‐CoA dehydrogenase (LCHAD, OMIM #201475) and very‐long‐chain acyl‐CoA dehydrogenase deficiency (VLCAD, OMIM #609016) [37]. Our panel's responses mirror this, with a similar number of experts (23%) deeming carnitine essential for both LCHAD and VLCAD, while a larger proportion (37%) disagreed. Such variability in clinical practice may be influenced by factors such as the rarity of IMDs, varied disease progression, and the limited availability of high‐quality evidence and guidelines [38, 39, 40].

Although this study was conducted within a European regulatory context, similar barriers to patient access, including regulatory ambiguity and limited reimbursement, have been reported for (s)NTPs in the United States [1, 21, 41]. In low‐ to middle‐income countries (LMICs), access can be even more constrained, as the high cost of importing (s)NTPs combined with the absence of reimbursement renders these treatments unaffordable for many patients [42, 43, 44]. Given their relative affordability compared to innovative cell‐ or gene‐based therapies, sNTPs may represent a more accessible treatment option in resource‐limited settings. While a separate, targeted list of essential sNTPs may be the most practical solution within the European context, from a global perspective their incorporation into the WHO essential medicines list may be justified to support broader access to these treatments, particularly in LMICs.

## 5 Limitations

Although this study combined expert consensus with a review of the literature for each sNTP, it is important to recognize that the underlying evidence base remains limited at Levels 3–4. This limitation reflects a broader challenge in the field of (nutritional) therapy and IMD management. The rarity and heterogeneity of IMDs means randomized controlled trials (RCTs) are often infeasible, and therefore many IMD therapies and guideline recommendations regarding their use are based on case reports, small studies, or expert opinion [38, 40, 45, 46]. Additionally, the literature search was structured and targeted, but non‐systematic. Given the breadth of 165 sNTP–IMD combinations reviewed, a fully systematic search for each combination was not feasible within the scope of this study, and therefore some relevant publications for individual sNTPs may not have been identified.

A proportion of panelists reported no specific expertise for several ultra‐rare IMDs, which may have affected the level of certainty in the evaluation of these conditions. Although the panel was composed to ensure broad coverage across metabolic disorders, complete specialist representation for all ultra‐rare conditions was not achievable. Future studies may benefit from the use of targeted sub‐panels with condition‐specific expertise to strengthen assessments in these areas.

Furthermore, the panel consisted exclusively of metabolic physicians. While this ensured disease‐specific clinical expertise, the absence of nutrition specialists limits multidisciplinary appraisal of the findings. Finally, the iterative consensus process did not include controlled feedback of group responses between rounds. As such feedback can facilitate convergence of expert opinions, its absence may have limited further refinement of consensus.

This list of essential sNTPs also does not account for the current variability in registration status. Certain sNTPs, such as pyridoxine, are already authorized as medicines, although reimbursement can remain challenging and impose a financial burden on patients [47].

## 6 Future Perspectives

In order to best address unmet therapeutic needs, future research should focus on identifying sNTPs with the most significant access challenges and evaluate the most effective routes to improve their availability. Authorizing them as medicines may help but does not automatically resolve all availability or affordability issues, especially since their price will likely increase. This can then be a new roadblock to patient access. For example, while authorized products for UCDs appear to have more reliable availability than unauthorized therapies, their high cost was found to impede access [48]. Nonetheless, clinicians clearly value formal authorization: a recent survey by Stepien et al. of healthcare professionals managing UCDs found that all respondents preferred licensed products, emphasizing their consistent supply, easier reimbursement, and the protections offered by regulatory oversight [49].

Beyond expert opinion, future efforts should also incorporate patient and caregiver perspectives, which this study did not capture. Especially in the development of new pharmaceutical formulations of sNTPs for authorization, patient perspectives on aspects such as tolerability and palatability are vital. Bringing together expert and patient perspectives helps ensure that this list of essential sNTPs translates into improved access and suitability, addressing the most pressing unmet needs.

## Author Contributions

C.E.M.H. and N.N.S. conceptualized the review. N.N.S. extracted the initial dataset. Data analysis, including evidence grading, was performed by N.N.S. and B.M.F.P. with subsequent refinement supported by C.E.M.H. The analytical process and data interpretation were also reviewed by A.M.B., M.L., and G.S.S. N.N.S. drafted the original manuscript. B.M.F.P. prepared Figure 2. C.E.M.H. supervised the project. Members of the “Essential nutritional therapy products for IMDs consensus group” participated in the Delphi process, and contributed expert input to the assessment and interpretation of the data. All authors reviewed, revised, and approved the final manuscript.

## Funding

This work was supported by the Postcodeloterij.

## Ethics Statement

The authors have nothing to report.

## Conflicts of Interest

C.E.M.H. is a member of the advisory committee to the insured healthcare package (Dutch Healthcare Institute), member of round table on orphan drugs (Dutch Healthcare Institute), executive member of the RARE‐NL foundation and chair of “Medicines for Society” (Medicijn voor de Maatschappij). Medicines for Society is an academic initiative that aims to support sustainable access to medicines for rare diseases, including NTPs. N.N.S. and B.M.F.P. are members of RARE‐NL and Medicines for Society. The remaining authors declare no conflicts of interest.

## Supporting information


**Supporting Information: Files S1.** List of sNTP–IMD combinations assessed in the literature review and in both Delphi rounds, including the Delphi results.

**Supporting Information: Files S2.** Modified Delphi survey questionnaire.

## Data Availability

The authors confirm that the data supporting the findings of this study are available within the article and its Supporting Information.
