# Supplementary material for: Essential Oral Single Nutritional Therapy Products for Inherited Metabolic Diseases: Evidence and Consensus Assessment Using a Modified Delphi Method
Source: J Inherit Metab Dis. 2026 Sep 30;49(6):e70252. doi: 10.1002/jimd.70252 (PMC13624829; doi:10.1002/jimd.70252)
Supplement: Supplementary file 1 [file JIMD-49-0-s002.docx]

**Supplementary Files S2: Modified Delphi survey questionnaire**

Survey hosted on the online platform eDelphi.org

**1. Essential nutritional therapy products in IMD management: ROUND 1**

Page 1 (1/20)

**Introduction**

**Background: nutritional therapy**

Nutritional therapy is critical in treating many inherited metabolic diseases (IMDs). It can involve various approaches, including specialized diets, low-protein foods, and oral single nutritional therapy products (NTPs, see definition below). Examples of NTPs are single amino acids, vitamins or cofactors. NTPs are frequently regulated as food or food supplements, rather than as medicinal products – even if they *function* as medicine for a specific IMD. This classification can result in challenges in patient access due to factors like inadequate reimbursement and less stringent regulatory oversight, meaning quality and dosing precision can vary.

In order to improve access to the NTPs required for adequate IMD treatment, there is a growing need to recognize NTPs that are essential for managing these conditions. The WHO defines essential medicines as those that satisfy the priority health care needs and should be available at all times, within functioning health systems, in adequate amounts, in appropriate dosage forms, with assured quality, and at affordable prices. This concept, when applied to IMDs, emphasizes the need for certain NTPs to be classified as essential in order to improve the availability, affordability and suitability of critically important NTPs.

This study therefore aims to identify the NTPs that are essential to IMD management.

**Defining NTPs**
This study focuses on **oral single nutritional therapy products (referred to as NTPs**). We defined NTPs as single nutritional therapy products, and therefore diets and amino acid mixtures fall outside the scope of this study. Additionally, as this study is focused on food products that function as medication in IMD management, only products for oral consumption were considered. Non-oral products are excluded as they do not classify as food.

Page 2 (2/20)

**Identifying NTPs utilized in IMD management: methodology**

**1. Creating a database**

To create a complete list of NTPs used in treating IMDs, the textbook *Inborn Metabolic Diseases: Diagnosis and Treatment* (7th edition, 2022) was used as the main source. A database was created listing all IMDs from the textbook, and the NTPs used in their treatment were extracted. Each NTP's role in managing an IMD was evaluated separately, resulting in 152 NTP and target IMD combinations.

**2. Identifiying potentailly essential NTPs**

To identify NTPs with the potential for essential classification, we then examined their functioning in IMD management. This process involved assessing the therapeutic intent, evidence base, physiological impact, dosing, and monitoring requirements of each NTP. These elements are based on our previously published flowchart(2) for distinguishing between food and medication-like functioning of NTPs.

The goal was to isolate those NTPs that demonstrate characteristics akin to medicinal products, thereby justifying their consideration as essential for managing IMDs. Data was gathered through PubMed searches for each NTP and target IMD.

**3. Inclusion in the modified Delphi procedure**

NTPs with (a) at least some observational evidence for their use in the target IMD (classified as ≥ level 4 evidence (3)) and (b) that met criteria for medication-like functioning were included in the Delphi process. A total of x NTPs and their target IMDs were included for consideration by the Delphi panel.

**More information?** For further details on the methodology and the assessment of each NTP, see the background documents provided on the eDelphi platform.

*References:*

1. Saudubray J.M. BMR, García-Cazorla A., Walter J. Inborn Metabolic Diseases: Diagnosis and Treatment. 7th edition ed: Springer Science & Business Media.; 2022.

2. Stolwijk NN, Bosch AM, Bouwhuis N, Häberle J, van Karnebeek C, van Spronsen FJ, et al. Food or medicine? A European regulatory perspective on nutritional therapy products to treat inborn errors of metabolism. J Inherit Metab Dis. 2023;46(6):1017-28. <https://onlinelibrary.wiley.com/doi/10.1002/jimd.12677>

3. OCEBM Levels of Evidence Working Group*. “The Oxford Levels of Evidence 2”. Oxford Centre for Evidence-Based Medicine. <https://www.cebm.ox.ac.uk/resources/levels-of-evidence/ocebm-levels-of-evidence>

Page 3 (3/20)

**How to participate in the eDelphi**

**eDelphi instructions**

In the following eDelphi survey, you will receive a series of NTPs reported in the management of a specific IMD to review. The IMDs are listed by disease group, which appear in alphabetical order.

- For each NTP, you can assess if you deem the product in question essential to managing the target IMD. You can click one of the  following options to provide your response: agree, neutral, disagree
- If an IMD falls outside your area of expertise, you have the option to opt out of responding to that particular combination.
- Additionally, you have the option to comment on each page.
- At the end of the survey, you will also have the opportunity to suggest NTPs that may have been missed.

Please select the option that best reflects your opinion. Your responses will help build consensus on the nutritional therapy products under review.

**Thank you for your participation.**

**Any questions or technical issues?** Please contact Nina Stolwijk at n.n.stolwijk@amsterdamumc.nl

Page 4 (4/20)

*Notes: OMIM codes were hyperlinked to their respective OMIM entries. See part 3 of these supplementary files and Supplementary Files S1 for the background documents.*

Page 5 (5/20)

Page 5 (5/20)

Page 6 (6/20)

Page 7 (7/20)

Page 8 (8/20)

Page 9 (9/20)

Page 10 (10/20)

Page 11 (11/20)

Page 12 (12/20)

Page 13 (13/20)

Page 14 (14/20)

Page 15 (15/20)

Page 16 (16/20)

Page 17 (17/20)

Page 18 (18/20)

Page 19 (19/20)

**Suggestions for NTPs**

Please let us know if there is any NTP you feel should be added to our list (please add the nutritional therapy product and the target IMD):

Page 20 (20/20)

**End of the eDelphi**

Thank you for your participation.

If you have any remarks or technical issues, please contact Nina Stolwijk: n.n.stolwijk@amsterdamumc.nl

**2. Essential nutritional therapy products in IMD management: ROUND 2**

Page 1 (1/9)

**Welcome to the second round of the Delphi procedure**

In this round, panelists will  have the opportunity to reassess NTPs carried forward from round 1 and review the newly suggested NTPs, considering feedback from the initial round. The goal of this phase is to refine consensus on which NTPs should be considered essential for IMD management.

**Summary of Delphi round 1** 
In the first round, 36 panelists assessed 82 NTPs and their associated IMDs (e.g., L-citrulline for CPS1 deficiency). Panelists evaluated whether each NTP was essential based on clinical experience and existing knowledge. The responses were then analyzed for consensus.

- NTPs with ≥75% agreement were classified as essential.
- NTPs with ≥50% disagreement were excluded as not essential.
- NTPs lacking consensus were carried forward to round 2 for re-evaluation.

Additionally, panelists suggested new NTPs that were not included in the initial assessment. These suggestions were reviewed, and those meeting predefined criteria for essentiality were included in round 2.

**Study design: brief reminder of key concepts**

**1. Backgound and objectives**

Nutritional therapy is essential in treating inherited metabolic diseases (IMDs), involving specialized diets, low-protein foods, and oral single nutritional therapy products (NTPs) such as amino acids, vitamins, and cofactors. NTPs are often regulated as food or supplements rather than medicines, leading to challenges in patient access due to variability in quality, dosing, and reimbursement. To improve access, there is a need to classify certain NTPs as essential, following the WHO's definition of essential medicines, which should be consistently available, of assured quality, and affordable. This study aims to identify the NTPs essential for effective IMD management.

**2. Defining NTPs**

This study focuses on oral single nutritional therapy products (referred to as NTPs). We defined NTPs as single nutritional therapy products, and therefore diets and amino acid mixtures fall outside the scope of this study. Additionally, as this study is focused on food products that function as medication in IMD management, only products for oral consumption were considered. Non-oral products are excluded as they do not classify as food.

Page 2 (2/9)

**Input for the Delphi procedure: round 2**

**NTPs included in round 2: methodology**

The second round of the Delphi procedure consists of 32 NTPs and their target IMDs. These  NTPs were included in round 2 from two sources: (1) NTPs that lacked consensus in round 1 and required further evaluation, and (2) new NTPs suggested by panelists, which were assessed for their potential essentiality using the same criteria as those in the initial round.

**1. NTPs without consensus after round 1**

NTPs lacking a clear consensus in round 1 are re-evaluated in round 2 (n = 18). Panelists are invited to reassess these NTPs, considering feedback from the first round.

**2. NTPs suggested in round 1**

Additionally, during round 1, the panel proposed NTPs not originally included. To identify suggested NTPs with the potential for essential classification, we examined their functioning in IMD management following the same methodology as the NTPs included in the first round. This process involved assessing the therapeutic intent, evidence base, physiological impact, dosing, and monitoring requirements of each NTP. These elements are based on our previously published flowchart(1) for distinguishing between food and medication-like functioning of NTPs.

The goal was to again isolate those NTPs that demonstrate characteristics akin to medicinal products, thereby justifying their consideration as essential for managing IMDs. Data was gathered through PubMed searches for each NTP and target IMD. NTPs with (a) at least some observational evidence for their use in the target IMD (classified as ≥ level 4 evidence (2)) and (b) that met criteria for medication-like functioning were included in the second round of the Delphi procedure (n=14).

More information? For further details on the methodology and the assessment of each NTP, see the background documents provided on the eDelphi platform.

*References:*

1. Stolwijk NN, Bosch AM, Bouwhuis N, Häberle J, van Karnebeek C, van Spronsen FJ, et al. Food or medicine? A European regulatory perspective on nutritional therapy products to treat inborn errors of metabolism. J Inherit Metab Dis. 2023;46(6):1017-28. <https://onlinelibrary.wiley.com/doi/10.1002/jimd.12677>

2. OCEBM Levels of Evidence Working Group*. “The Oxford Levels of Evidence 2”. Oxford Centre for Evidence-Based Medicine. <https://www.cebm.ox.ac.uk/resources/levels-of-evidence/ocebm-levels-of-evidence>

Page 3 (3/9)

**How to participate in the eDelphi**

**eDelphi instructions**

In the following eDelphi survey, you will receive a series of NTPs reported in the management of a specific IMD to review. The IMDs are listed by disease group, which appear in alphabetical order.

- For each NTP, you can assess if you deem the product in question essential to managing the target IMD. You can click one of the  following options to provide your response: agree, neutral, disagree
- If an IMD falls outside your area of expertise, you have the option to opt out of responding to that particular combination.
- Additionally, you have the option to comment on each page.
- At the end of the survey, you will also have the opportunity to suggest NTPs that may have been missed.

Please select the option that best reflects your opinion. Your responses will help build consensus on the nutritional therapy products under review.

**Thank you for your participation.**

**Any questions or technical issues?** Please contact Nina Stolwijk at n.n.stolwijk@amsterdamumc.nl

Page 4 (4/9)

*Notes: OMIM codes were hyperlinked to their respective OMIM entries. See part 3 of these supplementary files and Supplementary Files S1 for the background documents.*

Page 5 (5/9)

Page 6 (6/9)

Page 7 (7/9)

Page 8 (8/9)

Page 9 (9/9)

**End of the eDelphi**

Thank you for your participation.

If you have any remarks or technical issues, please contact Nina Stolwijk: n.n.stolwijk@amsterdamumc.nl

**3. Background documents**

Page 1 (1/5)

**1. Background**
Nutritional therapy is critical in the treatment of many inherited metabolic diseases (IMDs). It can involve various approaches, including specialized diets, low-protein foods, and oral single nutritional therapy products (NTPs, see definition below).^1^ Examples of NTPs are single amino acids, vitamins or cofactors (e.g. L-citrulline in carbamoyl phosphate synthetase 1 (CPS1) deficiency). NTPs are frequently regulated as food or food supplements, rather than as medicinal products – even if they function as medicine for a specific IMD.^2^ L-citrulline, for instance, is categorized as pharmacotherapy for the long-term management of CPS1 deficiency by relevant guidelines.^3^ Regulating NTPs as food can result in challenges in patient access due to factors like inadequate reimbursement and less stringent regulatory oversight, , which can lead to variations in quality and dosing accuracy.

The WHO defines essential medicines as those that satisfy the priority health care needs and should be available at all times, within functioning health systems, in adequate amounts, in appropriate dosage forms, with assured quality, and at affordable prices.^4^ Medicinal products that were deemed essential by the WHO have a higher global availability as compared to drugs that were deemed non-essential.^5^

This concept, when applied to IMDs, emphasizes the need for certain NTPs to be classified as essential in order to improve their availability, reimbursement and suitability (being quality and dosing precision). This study therefore aims to identify the NTPs that are essential for adequate IMD management.

**References**

1. Camp KM, Lloyd-Puryear MA, Huntington KL. Nutritional treatment for inborn errors of metabolism: indications, regulations, and availability of medical foods and dietary supplements using phenylketonuria as an example. Mol Genet Metab. Sep 2012;107(1-2):3-9. doi:10.1016/j.ymgme.2012.07.005
2. Stolwijk NN, Bosch AM, Bouwhuis N, et al. Food or medicine? A European regulatory perspective on nutritional therapy products to treat inborn errors of metabolism. J Inherit Metab Dis. Nov 2023;46(6):1017-1028. doi:10.1002/jimd.12677
3. Häberle J, Burlina A, Chakrapani A, et al. Suggested guidelines for the diagnosis and management of urea cycle disorders: First revision. J Inherit Metab Dis. Nov 2019;42(6):1192-1230. doi:10.1002/jimd.12100
4. Organization WH. Essential medicines. https://www.who.int/westernpacific/health-topics/essential-medicines#tab=tab_1
5. Bazargani YT, Ewen M, de Boer A, Leufkens HG, Mantel-Teeuwisse AK. Essential medicines are more available than other medicines around the globe. PLoS One. 2014;9(2):e87576. doi:10.1371/journal.pone.0087576

Page 2 (2/5)

**Methods**

**Definitions**
This study focuses on oral single nutritional therapy products (referred to as NTPs). Hence, diets and amino acid mixtures fall outside the scope of this study. Compounds already available as medicines, such as chenodeoxycholic acid, as well as non-oral products are excluded as the latter group does not classify as a nutritional therapy.

**Input for the Delphi procedure**
To compile a comprehensive list of NTPs used in the treatment of  IMDs, the standard textbook Inborn Metabolic Diseases: Diagnosis and Treatment (7th edition, 2022)^1^ was used as a primary source. A database encompassing all IMDs mentioned in this textbook was created. All NTPs described in their treatment were extracted. If multiple NTPs were described in the treatment of one IMD, each NTP was assessed separately this specific IMD.

To identify NTPs that may qualify as essential medicines, five elements from our previously published flowchart^2^ (see fig. 1, next page) for distinguishing between food and medication-like functioning of NTPs were assessed for each NTP and target IMD:

1) Purpose (to treat underlying metabolic defect)

2) Level of scientific evidence (using the Oxford Centre for Evidence-Based Medicine (OCEBM) scale^3^, see table on following pages)

3) The ability of the active ingredient to have a significant impact on physiological functioning

4) Dosing (supraphysiological or narrow therapeutic window)

5) Monitoring requirements.

To gather data for this evaluation, separate searches were executed in the PubMed database for each IMD-NTP combination.

**References**

1. Saudubray J.M. BMR, García-Cazorla A., Walter J. Inborn Metabolic Diseases: Diagnosis and Treatment. 7th edition ed. Springer Science & Business Media.; 2022.
2. Stolwijk NN, Bosch AM, Bouwhuis N, et al. Food or medicine? A European regulatory perspective on nutritional therapy products to treat inborn errors of metabolism. J Inherit Metab Dis. Nov 2023;46(6):1017-1028. doi:10.1002/jimd.12677
3. Group OLoEW. The Oxford Levels of Evidence 2. Oxford Centre for Evidence-Based Medicine. 2011;

Page 3 (3/5)

**Figure 1.** Proposed flowchart for distinguishing between food and medication-like functioning of nutritional therapy products.

Page 4 (4/5)

**Example: PLP in PNPO deficiency**

Pyridoxamine-5’--phosphate oxidase (PNPO) deficiency (OMIM #610090) is a rare inborn error of vitamin B6 metabolism that most often manifests as seizures during the neonatal period.^1,2^ These seizures can be managed effectively with vitamin B6 therapy, using either pyridoxine or its active form, pyridoxal phosphate (PLP). Without appropriate treatment, PNPO deficiency is fatal, with most affected children not surviving beyond their first year of life.

PLP (both forms) can be considered essential NTPs in the management of PNPO deficiency, as a subset of PNPO deficient patients present with seizures that only respond to PLP therapy.^1,2^ When applying the five criteria: (1) PLP addresses the underlying metabolic defect by restoring insufficient PLP concentrations caused by dysfunctional PNPO, (2) its effectiveness as anticonvulsive monotherapy is supported by case reports and case series, (graded as level 3, observational evidence graded up for (very) strong effect), (3) its significant physiological impact in suppressing seizures by restoring PLP levels is well-documented, (4) it is prescribed in supra-physiological dosages of 30 mg/kg/day, far exceeding the age-dependent vitamin B6 limit of 5–25 mg/day set by the European Food Safety Authority  (EFSA) and (5) careful monitoring of seizure control and liver function is required due to the potential for liver toxicity.  In order to adequately manage PNPO deficiency, it should be available at all times, in adequate amounts, in appropriate dosage forms, with assured quality, and at affordable prices.

**References**

1.  Alghamdi M, Bashiri FA, Abdelhakim M, et al. Phenotypic and molecular spectrum of pyridoxamine-5'-phosphate oxidase deficiency: A scoping review of 87 cases of pyridoxamine-5'-phosphate oxidase deficiency. Clin Genet. Jan 2021;99(1):99-110. doi:10.1111/cge.13843
2. Mills PB, Surtees RA, Champion MP, et al. Neonatal epileptic encephalopathy caused by mutations in the PNPO gene encoding pyridox(am)ine 5'-phosphate oxidase. Hum Mol Genet. Apr 15 2005;14(8):1077-86. doi:10.1093/hmg/ddi120

Page 5 (5/5)

**The modified Delphi approach**

**Expert panel composition**
Metabolic physicians with broad clinical experience are invited to participate in the expert panel through the emailing list of  the Society for the Study of Inborn Errors of the Metabolism (SSIEM). Additionally, personal invitations will be sent to metabolic experts for each IMD group, because of their expert knowledge based upon their publication track record.

**Delphi procedure overview**
NTPs with at least some observational evidence for their functioning in an IMD (classified as ≥ level 4 evidence) that meet all five flowchart criteria are included in the Delphi process. The aim is to assess if the metabolic expert panel, drawing on their own clinical experience, deems these NTPs essential to the treatment of the specific IMD. This process, conducted through a modified Delphi procedure, does not require panelists to revisit or reanalyze existing literature.

Panelists will be provided with a structured questionnaire listing all eligible NTPs and their target IMDs (n=82) via the web-based software tool eDelphi (https://www.edelphi.org/). For each NTP and target IMD (e.g. L-citrulline in CPS1 deficiency), panelists will assess whether the NTP is essential for managing the specific IMD, using a three-point Likert scale (agree, neutral, disagree). Panelists will also have the option to indicate if they have no expertise on a particular disease. In addition, there is the option to leave comments.

After a two-week response period for the first round, the responses will be analyzed to assess consensus. NTPs with ≥75% agreement will be deemed essential for their target IMD and included in the final list. Combinations with ≥50% disagreement will be excluded as  “not essential”. Panelists who indicated that they have no expertise on a particular disease will be excluded from the analysis of that specific NTP and target IMD. Combinations that were not agreed or disagreed upon in the first round will enter a second round, where panelists will have the opportunity to reassess their responses, using the feedback and comments from the first round to refine the consensus.

After the second round, combinations with ≥75% agreement will be included in the final list, while combinations with ≥50% disagreement will again be excluded as  “not essential”. Combinations with less than 75% agreement and less than 50% disagreement will be categorized as “no consensus”. These will be excluded from the final list of essential NTPs and published as a separate list.

In addition, the results of the structured literature search included in Supplementary Files S1 were provided.
